# Proximity Bias Following Affective Metaphors in Patients With Depression—Psychoanalytic Considerations

**DOI:** 10.3389/fpsyg.2019.02438

**Published:** 2019-11-06

**Authors:** Iftah Biran, Assaf Tripto, Anat Arbel

**Affiliations:** ^1^Division of Psychiatry, Chaim Sheba Medical Center, Ramat Gan, Israel; ^2^Neurological Institute, Tel Aviv Sourasky Medical Center, Tel Aviv, Israel; ^3^The Israeli Neuropsychoanalysis Society, Kadima, Israel; ^4^Sackler Faculty of Medicine, Tel Aviv University, Tel Aviv, Israel; ^5^Beeri Clinic, Kupat Holim Clalit, Bnei Brak, Israel

**Keywords:** depression, metaphors, embodiment, line bisection, spatial attention

## Abstract

**Background:** Many languages use spatial metaphors to describe affective states such as an upward bias to denote positive mood, a downward bias to denote negative mood, a body proximity bias to denote personal relatedness concern, and a right-left bias to denote negative or positive valence. These biases might be related to experiential traces related to these affective states. If this is the case, depressed subjects would show either a downward spatial bias, a body proximity bias, or a right-left shift in attention. We evaluated the occurrence of such biases in subjects with depression compared to healthy controls.

**Methods:** *Subjects*: 10 subjects with depression (5F:5M; age = 47.2 ± 15.2) and 10 healthy controls (5F:5M; age = 45.8 ± 14.5). *Experimental task*: line bisection task. Lines were presented in three spatial orientations [vertical (up-down), horizontal (right-left), radial (proximal-distal)] and were either blank, composed with words (negative/positive/neutral), or with smileys (negative/positive/neutral). There were 21 line types, and each was presented eight times, reaching a total of 168 lines.

**Results:** Compared with healthy controls, subjects with depression bisected radial lines significantly closer to their body. There were no significant differences for either horizontal or vertical lines.

**Conclusion:** The proximity spatial bias observed in subjects with depression suggests that depression might activate neural spatial networks. We argue that these networks could be dynamically activated through narcissistic mechanisms as implied in “Mourning and Melancholia” where Freud postulates a narcissistic mediated bias in depression according to which the depressed subjects withdraw from the outside world.

## Introduction

Many languages convey emotional states through spatial metaphors, such as the spatial “*up-down”* metaphor where “down” denotes sad or depressed mood, whereas “up” denotes good mood to the extent of manic states.

These spatial metaphors are common in our daily use (Lakoff and Johnson, 2003b): In the negatively charged downward direction: “depressed mood,” “feeling down,” “he is really low,” “he fell into depression,” and “my spirits sank,”, and in the opposite direction: “elevated mood,” “feeling high,” “that boosted my spirits,” and “my spirits rose,” Similar metaphors are found across different cultures and multiple languages [e.g., in German – “in Stimmungstief sein” (to be in a depressed mood), “in gehobener Stimmung sein” (to be in high spirits); in French – “une baisse d’humeur” (low mood), “montée de l’humeur” (increase in the mood); and in Hebrew – “*matzav ruach meromam”* (elevated mood), “*matzav ruach yarud*” (depressed mood)].

This metaphoric vertical spatial encoding of affective states is also encountered in literature and poetry. Emily Dickinson brings in her poem “*I felt a Funeral, in my Brain*”, a horrifying description of depression illustrated as a never ending fall – “And then a Plank in Reason, broke,/And I dropped down, and down – /And hit a World, at every plunge,/And Finished knowing – then –”(Dickinson, 1983). This depressive downward fall fits the common western metaphors of depression as falling down and descent (Pritzker, 2003; McMullen and Conway, 2014).

Spatial affective metaphors are not limited to the vertical up-down axis and can be extended to a horizontal right-left axis where right is regarded as good and left is regarded as bad (Drago et al., 2010) or to a horizontal toward–away/distal-proximal (radial) axis – as in “it touched my heart,” “it touched me,” and “I am going to leave it behind my back,” all of which are used to denote an affective personally loaded state and can shed light on the individual’s personality style (Hastings, 1952; Fisher, 1964).

Are these “real” metaphors in the sense that they entail symbolic idiomatic thinking or are they mere concrete embodiment of the affective state?

In order to address this, we first have to look at the definition of metaphors. Classically, metaphors are regarded as a figurative act of speech in which a “thing” from one domain is used to describe a second “thing” from a different domain or as “the cognitive mechanism whereby one experiential domain is partially ‘mapped,’ i.e., projected, onto a different experiential domain, so that the second domain is partially understood in terms of the first one” (Barcelona, 2000). The definition of metaphors in the Merriam-Webster dictionary is similar: “a word or phrase denoting one kind of object or action is used in place of another to suggest a likeness or analogy between them.”^1^ In this regard, a metaphor is a tool used to suggest or direct the addressee to a rather obscure and hidden similarity between two entities from different domains, and in order to understand the metaphor, one should find out what is depicted by the metaphor and what quality is transposed between the two domains.

Lakoff and Johnson in their book “Metaphors We Live By” use a definition that at first hand seems similar to the above-quoted classic definitions but is quite different and signifies a revolution in the study of metaphors their definition goes as follows: “The essence of metaphors is understanding and experiencing one kind of thing in terms of another” (Lakoff and Johnson, 2003a, p. 5). This combination of both understanding and experiencing suggests that a metaphor is both a cognitive entity and a symbolic entity and that it stems from both understanding through a cognitively mediated process as well as from an experiential act that is felt directly as the thing itself.

Going back to spatial affective metaphors: The question whether affective metaphors are a figure of speech mediated by linguistic cognition or an experiential phenomenon is related to a clinical issue – that of symbolization charged with unconscious meaning as opposed to (or differing from) concrete thinking and experiences. When a patient uses affective metaphors in an encounter with his or her therapist, can these be reduced (or expanded) to a covert underlying meaning that is exposed through analytic work? Or rather that these spatial metaphors express bodily sensations and are the experience itself? This carries within clinical implications as the former directs to classic psychoanalytic work emphasizing on the verbal while the latter directs to bodily based techniques emphasizing on the non-verbal and on embodied attentiveness (Bloom, 2000, 2018).

If affective spatial metaphors are indeed experiential, we can expect a spatial bias in affective loaded conditions. This is in accordance with the Hebbian postulate that “Neurons that fire together wire together” (Hebb, 1949)^2^, and in this context, affective networks that co-activate with certain body-spatial networks synapse together.

Previous research demonstrated this spatial-affective interaction mainly in healthy non-depressed subjects. Most of the studies looked at just one axis (either vertical, horizontal, or radial) and did not address the various axes using a single paradigm.

Drago et al. demonstrated that subjects tended to place a mark on a blank sheet of paper according to an affective state induced experimentally. On a radial-vertical axis (going from higher-distal to lower-proximal), healthy subjects marked the page more distally and higher following happy conditions and more proximal and lower following sad conditions (Drago et al., 2010). Foster et al. demonstrated using a similar paradigm that healthy subjects placed pegs labeled with various emotions differentially on a board. Positively labeled pegs were placed higher and distally (Foster et al., 2008). In another study, exposure to happy music activated a rightward bias in visuospatial horizontal line bisection, whereas exposure to sad music activated an opposite bias (Barrow, 2013). Fisher demonstrated that healthy subjects in a sad mood, as judged by the way they described faces, tended to have a preference for downward movements and while adjusting rods in a horizontal position tended to err downward (Fisher, 1964).

Surprisingly, although there is an extensive research regarding general non-directional spatial attentional impairments in depressed subjects [see, e.g., the study by Ossowski et al. (2011)], there is almost no research regarding specific directional spatial bias in depressed population. Using tachistoscopic presentation of happy-sad chimeric face drawings, David et al. were able to show that while healthy controls and manic patients tend to have a leftward attentional bias (they tend to perceive the left hemi-face), depressed patients do not show this effect (David, 1993). Studies looking at the performance of depressed subjects bisecting horizontal lines presented on the right-left axis were inconclusive (Cavezian et al., 2007; Ramos-Brieva et al., 2009; Wei et al., 2010). To the best of our knowledge, there are no studies that looked at the vertical or the radial axes in subjects with depression.

The notion that spatial affective language is at times not figurative, but rather concrete is supported not only by experimental studies but also by observations on healthy subjects, clinical observations, and observations in psychodynamic therapy.

Canales et al. showed that depressed patients, in accordance with a downward bias, show a kyphotic flexed position of their trunk and that this position is normalized upon recovery (Canales et al., 2010). Similarly, subjects with symptoms of depression tend to be in an inclined protruded posture (Rosario et al., 2014). Reading sentences with themes of depression facilitated downward movement of healthy subjects (Santana and de Vega, 2011).

This can also be encountered in images, dreams, and associations brought to therapy by depressed patients (McMullen and Conway, 2014). Here are illustrative clinical examples: (1) an elderly man presenting with psychotic depression who describes how he is buried alive down under a tombstone, sand, and earth. The spatial distortion evident in this example is a vertical downward bias. (2) A young woman with depressive disorder and defensive grandiose defenses brings a dream in which she is transported from a cave like crypta of an underground city toward a high-rise. This journey is facilitated through a travel in a cable car that travels both in a horizontal forward direction and a vertical upward direction. This dream that was interpreted as a flight from her depression brings together both the vertical and the horizontal – radial axes. (3) Horizontal right-left bias could be the source for the higher prevalence of left sided hemiplegic conversion suggesting negative connotations and valence attributed to the left side. However, this finding is questionable and could be related to reporting bias (Stone et al., 2002).

Previous research and clinical observations did not compare the relative deviations of (or impairments to) the different axes. However, psychodynamic formulations are in accordance with the significance of an operation on the radial axis as an emblem of an inside-outside gradient. Such is the narcissistic formulation of depression as conceptualized by Freud. Freud suggests that depression is an inward withdrawal from the outside world to the inner world, accompanied by decathexis of real-world objects (Freud, 1917[1915]).

Do the above formulation as well as the spatial metaphors of depression have embodied correlates? In this study, we compare the spatial bias of depressed patients with that of healthy controls across all three axes [vertical (up-down), horizontal (right–left), radial (proximal-distal)]. We adapted the line bisection task that is usually used to test horizontal (right-left) spatial bias in patients with neglect following right hemispheric injury. These patients tend to neglect the left side of the space and to bisect horizontal lines in a position that is located right to the midline (Schenkenberg et al., 1980). As the same stimuli (lines) could be presented in different orientations while keeping the same visual characteristics, this task could be used to compare performance across all axes.

We hypothesize that subjects with depression would show a specific spatial bias in each of the axes – a leftward deviation in bisecting horizontal lines, a downward deviation in bisecting vertical lines, and a proximal deviation bisecting radial lines. We further hypothesized, based on the dynamic formulations of narcissistic bias in depression, that the deviation would be more prominent in the radial condition.

## Methods

### Subjects

Depression group (DEP): 10 adult subjects with depression were recruited from the outpatient psychiatric clinic of the Chaim Sheba Medical Center at Tel Hashomer. Depression diagnosis was based on clinical interview by a certified psychiatrist, and they all met DSM-5 criteria for depression (American Psychiatric Association, 2013). Patients were treated with either anti-depressants, individual psychotherapy, group psychotherapy, or a combination. Control group (CON): 10 sex- and age-matched control subjects were recruited by the research team using snowball sampling and direct recruitment.

### Ethics

The study was approved by the Institutional Review Board (Helsinki Committee) of the Chaim Sheba Medical Center at Tel Hashomer.

### Baseline Characteristics

Cognitive functions were evaluated by The Neurobehavioral Cognitive Status Examination Revised 2011 (Cognistat) that enables the assessment of various cognitive domains (Kiernan et al., 1987; Katz et al., 1996; Cognistat, 2011). Depression was evaluated by filling out the Beck Depression Inventory-II (BDI-II) (Gil and Gilbar, 2001; Steer and Beck, 2001).

### Experimental Task

**Figure 1:**
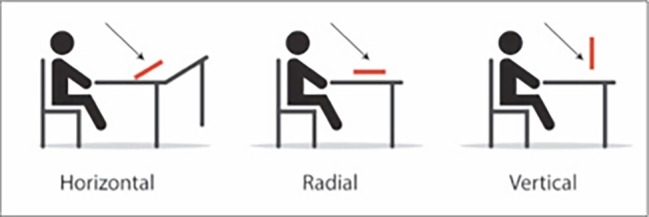
The three line orientations (axes).

**Figure 2:**
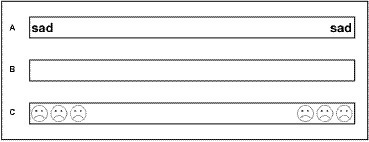
Illustration of the various line types. **(A)** Horizontal negative word line; **(B)** Horizontal blank line; **(C)** Horizontal negative smiley line.

The experimental task was based on the line bisection task that is used to assess hemi-spatial neglect. In this task, the subject is asked to bisect a line in its midline (Schenkenberg et al., 1980). A deviation from the midline suggests an attentional bias. Lines were administered in three axes: vertical (up-down), horizontal (right-left), and radial (proximal-distal) (see Figure 1). Lines were 10 cm long and 0.5 cm wide. There were seven line types: a blank line; three line types with word cues at their ends: happy, sad, and neutral words; and three line types with visual affective cues at their ends: happy, sad, and neutral smileys (see Figure 2 for an example of the different line types). Each line type was presented eight times in each axis. In total, each subject was asked to bisect 168 lines [21 conditions (3 axes × 7 line types) × 8 repetitions for each condition]. Following the method used by Schenkenberg et al. (1980), all 8 repetitions of each of the 21 conditions were printed on an A4 paper with a distance of 1.8 cm between each line and were presented randomly at a distance of 30 cm from the subjects. The measurements were done using ABSOLUTE Digimatic Caliper with an accuracy of 0.001 cm (Mitutoyo, 2016). The point of bisection was measured twice for each line, and the two measurements were averaged. The measurements were as follows: the distance from the left for horizontal lines, the distance from the top for vertical lines, and the distance from the distal end for the radial lines.

### Statistical Analysis

Data were entered and analyzed using SPSS version 24. Descriptive statistics were produced using means and standard deviations for continuous variables (e.g., age), and frequencies for categorical variables (e.g., sex). To examine differences between groups in continuous variables, *t*-tests were conducted. To examine differences between groups in categorical variables, Chi-Square tests were conducted. Variables were tested for normal distribution using Kolmogorov-Smirnov procedures. To test study hypotheses, multivariate analysis of covariance (MANCOVA) was conducted. The level of significance is 5%.

## Results

### Baseline Characteristics

Participants in both groups were matched for gender, age, and education, and there was no significant difference between the two groups across these variables (see Table 1).

**Table 1:** Demographic and baseline characteristics.

	Control	Depression	Difference between groups
**Demographics**			
*N* (male/female)	10(5/5)	10(5/5)	*χ*^2^ = 1, *p* = 1.00
Age	45.8 ± 14.5	47.2 ± 15.3	*t* = 0.21, *p* = 0.83
Education [years]	15.3(2.4)	15.0 (2.7)	*t* = 0.26, *p* = 0.80
**Depression**			
BDI-II score	3.1 (2.1)	25.1 (5.64)	*t* = 11.56, *p* < 0.001
**Cognistat domains (normal range)**			
Orientation (9–12)	12.00 (0.00)	11.70 (0.48)	*t* = 1.96, *p* = 0.06
Attention (6–8)	7.80 (0.42)	7.70 (0.67)	*t* = 0.397, *p* = 0.69
Language – comprehension (5–6)	5.90 (0.31)	5.90 (0.31)	*t* = 0.000, *p* = 1.00
Language – repetition (10–12)	12.00 (0.00)	12.00 (0.00)	*t* = 0.000, *p* = 1.00
Language – naming (6–8)	7.50 (0.70)	6.50 (1.58)	*t* = 1.82, *p* = 0.08
Building (4–6)	5.90 (0.31)	5.10 (1.37)	*t* = 1.799, *p* = 0.08
Memory (9–12)	9.10 (2.99)	10.00 (3.36)	*t* = 0.631, *p* = 0.53
Calculia (3–4)	4.00 (0.00)	3.60 (0.96)	*t* = 1.309, *p* = 0.20
Similarities (5–8)	7.90 (0.31)	7.80 (0.63)	*t* = 0.447, *p* = 0.66
Decision (4–6)	6.00 (0.00)	5.60 (1.26)	*t* = 1.000, *p* = 0.343

*The BDI-II scores are as follows: 0–13, minimal depression; 14–19, mild depression; 20–28, moderate depression; 29–63, severe depression*.

As expected, average BDI-II was significantly higher among participants who were diagnosed with depression in comparison with the control group (25.10 ± 5.64 vs. 3.1 ± 2.07, respectively, *p* < 0.001) (see Table 1).

Comparison of the various cognitive domains between groups indicated no significant differences between groups across all cognitive domains (see Table 1).

### Experimental Task

Prior testing the main hypothesis, normality tests were conducted for all measures using Kolmogorov-Smirnov procedure. All measures were found with normal distribution (*p* of K-S > 0.60). Therefore, in order to test the differences between groups, MANCOVA was conducted.

Compared with healthy controls, subjects with depression bisected radial lines significantly closer to their body, suggesting a proximity spatial bias. This bias was consistently found for happy smiley lines (0.18 cm proximal, *p* = 0.03), sad smiley lines (0.21 cm proximal, *p* = 0.02), happy word lines (0.17 cm proximal, *p* = 0.05), and sad word lines (0.25 cm proximal, *p* = 0.02). There were no significant differences for either horizontal or vertical lines (see Table 2).

**Table 2:** Deviations in bisection of radial lines (measurements are the distance from the distal end in centimeters. The midline is 5 cm, proximal deviation is more than 5 cm, and distal deviation is less than 5 cm).

Line types		Control	Depression	Difference between groups
Smiley lines	Happy	4.72 ± 0.17	4.90 ± 0.18	*F* = 5.38, *p* = 0.03
	Sad	4.71 ± 0.13	4.92 ± 0.22	*F* = 6.55, *p* = 0.02
	Neutral	4.73 ± 0.17	4.88 ± 0.17	*F* = 3.69, *p* = 0.07
Word lines	Happy	4.70 ± 0.18	4.87 ± 0.19	*F* = 4.33, *p* = 0.05
	Sad	4.70 ± 0.15	4.95 ± 0.27	*F* = 6.26, *p* = 0.02
	Neutral	4.77 ± 0.16	4.86 ± 0.16	*F* = 1.36, *p* = 0.25
Blank line	—	4.76 ± 0.17	4.88 ± 0.12	*F* = 2.79, *p* = 0.11

## Discussion

To conclude, in this study, we were able to show that subjects with depression compared with normal controls tend to bisect lines presented in the radial axis with a proximal bias. That is, they tend to bisect radial lines significantly closer to their body compared with normal controls. This was observed for affective lines (happy and sad smileys, happy and sad words). No effect was observed for both horizontal and vertical lines.

Although this spatial bias is relatively small (up to 0.25 cm), it confirms our hypothesis regarding a proximity bias. The small effect size for the spatial bias is not surprising as similar miniscule effect sizes were observed previously (Wei et al., 2010). In any case, it could be secondary to the methodology employed in the study. The subjects were not asked to judge the valence and the emotionality of the stimuli, and therefore, this was addressed only implicitly. Other studies that looked at emotional stimuli explicitly demonstrated a bigger effect size (Drago et al., 2008; Foster et al., 2008).

As negative and positive stimuli can influence attention differentially (Baumeister et al., 2001; Yiend, 2010), it is surprising that the radial proximity bias was observed for both negative and positive words and smileys. This could be related to various parameters such as the arousal level of the stimuli or the depression as depression by itself can mask the differences between negative to positive stimuli (Beck, 1963, 1964).

The fact that the attentional bias documented in performing the line bisection tasks was confined to the radial axis is intriguing. As the tasks were similar across all axes, it is suggestive that this difference is related to specific and inherent mechanisms related to depression. This is in accordance with Churches et al. who demonstrated different cognitive mechanisms for vertical attention as opposed to horizontal attention (Churches et al., 2018).

Can the observed proximity bias be explained in psychoanalytic terms? Object relation theories, as originally formulated by Fairbairn, stress the importance of the infant’s need to relate to others. As the infant’s original objects, the mother and her breast, have distinct spatial coordinates, psychological processes such as differentiation and incorporation are intertwined with spatial operations (Fairbairn, 1941, 1963; Akhtar, 2009; Groarke et al., 2019). We argue that the proximity bias observed in depressed patients echoes this association between object relations and spatial coordinates. We further suggest that this bias follows (or mirrors) linguistic metaphors.

This is in accordance with Freud’s model of depression as outlined in “Mourning and Melancholia” where he claims that the melancholic withdraws from the outside world – “The distinguishing mental features of melancholia are a profoundly painful dejection, cessation of interest in the outside world” and that this process is related to decathexis of libido from an object in the outside world – “But the free libido was not displaced on to another object; was withdrawn into the ego” (Freud, 1917[1915]). The withdrawal follows narcissistic logic in the sense that in narcissistic operations subjects withdraw libidinal interest from the outside world onto the self. Thus, it can help in conditions with impoverished ego as the decathected libido, now bound to the depleted ego, enriches the ego and the self (Freud, 1914). Overall, this can serve as a defense against adverse object relations. The narcissistic object relationship protects the capacity to love as it makes the love – object seems like the self (Britton, 2004).

An alternative psychoanalytic model that explains this narcissistic spatial bias and withdrawal is the cannibalistic model of depression formulated by Karl Abraham. According to this model, depressed subjects internalize aggressions that are directed toward the outside world. Abraham stresses the role of aggression in the generation of depression, whereas an aggressive instinct is transmuted into depressive affect. The ambivalence toward the loved object and the wish to incorporate it takes the form of a cannibalistic action. The guilt feelings of the depressed are a reaction to his own cannibalistic oral desires (Abraham, 2002; Van Schoonheten, 2015).

Irrespective of the nature of what is being incorporated, whether it is an object that carries within ambivalent cathexis as formulated by Freud or aggressive representations as formulated by Abrams, our results suggest that the psychic incorporation is correlated with body and space coordinates.

The argument that objects’ relations are mapped spatially is supported, as stated above, by early developmental paradigms that base early object relations on achieving a spatial-bodily distinction between the baby and the world and others within the world. Such paradigms stress the early spatial interactions with the caregiver through a unique intermediate environment such as Rey’s “marsupial space” or Winnicott’s holding environment (Winnicott, 1960; Rey, 1988; McDougall, 1992; Allnutt, 2016).

Regardless of the specific psychoanalytic model, what is the neurophysiological basis of this depressive narcissistic withdrawal and of the decathexis of libido from an outside object to an internal representation? The right hemisphere could be a good candidate as it serves both the representations of the relationship between the self and objects and spatial cognition. Following right hemisphere damage, the distinction between self and objects is impaired and mature object relations are substituted by narcissistic relations, and this is accompanied by spatial distortions (Kaplan-Solms and Solms, 2002; Solms and Turnbull, 2002). It could be argued that a relative right hemispheric dysfunction (or dysregulation) in depression or right-left hemispheres misbalance is contributing to this clinical phenomenon (Bruder et al., 2017), and indeed, the right hemisphere is implicated in self-reflection, inward withdrawal, and introspection (Molnar-Szakacs et al., 2005; Hecht, 2010a,b). All these are commonly associated with depression (Höping et al., 2006).

Following all of the above and regarding the spatial-bodily metaphors in depression, we suggest that these metaphors are unique as they are not a mere transfer of meaning from one domain (affective) to another domain (space and body) but rather represent a distinct cognitive operation culminating in the observed spatial deviations. At times, as implied by Rene Magritte’s “Treachery of images,” “a pipe is just a pipe” and the signifier is the thing itself. However, it can still be understood through associations, experiencing, re-experiencing, and understanding.

To conclude, we suggest that this spatial proximity bias follows (or mirrors) linguistic metaphors. Based on psychodynamic formulations of depression, this proximity bias might be related to narcissistic mechanisms in depression, according to which depression is characterized by withdrawal from the outside world (Freud, 1917[1915]). Limitations of the current study are the small patient group and evaluation of the depression by psychiatric tools such as BDI-II depression questionnaire and the DSM-5 criteria without better psychoanalytic characterization. Although, there are no specific questionnaires used to characterize the narcissistic dimensions of depressed patients, the formulation of introjective depression versus anaclitic depression could be employed. According to this formulation, introjective depression has some narcissistic characteristic. Further research looking at this distinction is warranted (Blatt and Maroudas, 1992; McDougall, 1992; Blatt et al., 2001). Further research looking at the radial axis through other paradigms is warranted.

## Data Availability Statement

The datasets generated for this study are available on request to the corresponding author.

## Ethics Statement

The studies involving human participants were reviewed and approved by the Institutional Review Board (Helsinki Committee) of the Chaim Sheba Medical Center at Tel-Hashomer. The patients/participants provided their written informed consent to participate in this study.

## Author Contributions

All authors designed the study. AT and IB evaluated the patients. AA collected the data and organized the data set. IB analyzed the data. IB wrote the first draft of the manuscript. All authors contributed to manuscript revision, read, and approved the submitted version.

### Conflict of Interest

The authors declare that the research was conducted in the absence of any commercial or financial relationships that could be construed as a potential conflict of interest.

## Footnotes

^1^https://www.merriam-webster.com/dictionary/metaphor; Downloaded – 5/8/2019.

^2^The original quote from Hebb goes as follows: “When an axon of cell A is near enough to excite a cell B and repeatedly or persistently takes part in firing it, some growth process or metabolic change takes place in one or both cells such that A’s efficiency, as one of the cells firing B, is increased” (Hebb, 1949, p. 62).
